# Antigen-specific tolerance and control of autoimmunity effected by liver sinusoidal endothelial cells is unimpaired in liver fibrosis

**DOI:** 10.3389/fimmu.2026.1834595

**Published:** 2026-05-05

**Authors:** Cornelia Gottwick, Pia Averhoff, Christian Casar, Laura Anne Liebig, Sabrina Melanie Pilz, Victor Haas, Daria Krzikalla, Sabine Fleischer, Norbert Hübner, Lorenz Adlung, Dorothee Schwinge, Christoph Schramm, Antonella Carambia, Johannes Herkel

**Affiliations:** 1 Department of Medicine I, University Medical Center Hamburg-Eppendorf, Hamburg, Germany; 2 Hamburg Center for Translational Immunology (HCTI), University Medical Center Hamburg-Eppendorf, Hamburg, Germany; 3 Max Delbrück Center for Molecular Medicine in the Helmholtz Association (MDC), Berlin, Germany; 4 Topas Therapeutics GmbH, Hamburg, Germany; 5 Center for Biomedical AI (bAIome), University Medical Center Hamburg-Eppendorf, Hamburg, Germany; 6 Martin Zeitz Center for Rare Diseases, University Medical Center Hamburg-Eppendorf, Hamburg, Germany

**Keywords:** autoimmunity, immune tolerance, liver fibrosis, nanomedicine, scavenging

## Abstract

**Background and aims:**

Liver sinusoidal endothelial cells (LSECs) have a key role in maintaining organismal homeostasis by scavenging blood-borne molecules and inducing specific immune tolerance to ingested antigens. The scavenger and tolerance function of LSECs can be harnessed for specific treatment of autoimmune diseases by nanoparticle-mediated autoantigen delivery to LSECs. In liver fibrosis, which is a frequent condition in human populations, LSECs undergo changes promoting pro-fibrotic and pro-inflammatory activation of other hepatic cells, but it is unclear whether the scavenger and immune tolerance functions of LSECs are affected.

**Methods:**

Utilizing two mouse models of liver fibrosis, we explored the ability of LSECs to take up nanoparticles conjugated with antigen peptides, to present the ingested antigen peptides to T cells and to induce peptide-specific immune tolerance *in vitro* and *in vivo* in the context of autoimmune diseases.

**Results:**

LSECs from fibrotic livers showed few distinct adaptations regarding immune functions; however, overall LSEC identity was largely maintained. Accordingly, endocytosis of nanoparticles by LSECs *in vivo*, as well as processing and presentation of nanoparticle-bound antigen peptides was not compromised by liver fibrosis. LSECs from fibrotic livers maintained the ability to effectively induce the generation of regulatory T cells from conventional CD4 T cells. Hence, targeted delivery of autoantigen peptides to LSECs *in vivo* effectively induced specific tolerance despite liver fibrosis, providing protection in two models of experimental autoimmune disease. Analysis of datasets from human subjects with or without liver cirrhosis confirmed that scavenger and tolerance pathways in LSECs were preserved in human liver fibrosis.

**Conclusions:**

Scavenger activity and antigen-specific tolerance induction by LSECs are preserved in liver fibrosis. Thus, LSECs remain reliable mediators of homeostasis and tolerance under fibrotic conditions, and particularly suitable targets for nanomedicine products.

## 1 Introduction

Liver sinusoidal endothelial cells (LSECs) are specialized endothelial cells that separate the sinusoidal blood from the underlying liver parenchyma. Yet owed to cellular pores (fenestrae) and the lack of a basal membrane, LSECs form a permeable barrier that facilitates effective exchange of molecules between blood and hepatocytes. Moreover, LSECs feature unique capabilities for receptor-mediated endocytosis and scavenging of blood-borne waste molecules and turnover products both derived from gut microbiota or host cells (1, 2). Furthermore, LSECs can clear the circulation of small immune complexes, and of nano-sized viruses and particles (1, 2). These activities are facilitated by the expression of a variety of scavenger receptors combined with powerful endocytotic capacity (1, 2). Importantly, the ingested molecules and particles are processed to generate peptides that both can be presented to CD4+ T cells on MHC class II molecules or cross-presented to CD8+ T cells on MHC class I molecules, for the most part producing T cell tolerance (3–5). Of note, LSECs have a particular capacity to induce regulatory T cells (Treg) (4). This antigen-specific induction of T cell tolerance by LSECs is of vital importance for the host organism, as it can prevent potentially devastating chronic inflammation to abundant and harmless substances (3, 6). The downside of LSEC-induced antigen-specific tolerance is an increased risk for chronic infections and cancer, as LSEC-induced tolerance can also prevent effective elimination of infected (7) or malignant cells (8). Of note, the capacity of LSECs for antigen-specific tolerance induction can be harnessed therapeutically by LSEC-targeting nanoparticles that carry autoantigen peptides, providing induction of specific regulatory T cells *in vivo* and effective treatment of autoimmune diseases (9). Such nanomedicine can be used for tolerance induction both in CD4+ and CD8+ T cells (9–11) and is currently in clinical development (11, 12).

However, following chronic liver injury, LSECs dedifferentiate in a process called capillarization that leads to the loss of their characteristic fenestration, and the formation of a basal membrane. As a result, dedifferentiated LSECs acquire phenotypical and functional changes that contribute to pro-fibrotic and pro-inflammatory activation of other hepatic cells (13, 14). Whereas differentiated LSECs actively promote hepatic stellate cell quiescence, dedifferentiated LSECs permit stellate cell activation, thereby indirectly promoting fibrogenesis (15, 16). Moreover, dedifferentiated LSECs promote angiogenesis, vasoconstriction and increased leukocyte recruitment (17). Hence, liver fibrosis and cirrhosis are associated with LSEC dedifferentiation, whereas their redifferentiation seems to promote fibrosis regression (18).

To what extent the dedifferentiation of LSECs in fibrotic livers affects their scavenger and immune tolerance function is not entirely clear. On the one hand, it has been reported that dedifferentiated LSECs in liver fibrosis acquire enhanced immunogenicity, marked by increased production of pro-inflammatory mediators, increased antigen capture and enhanced T cell activation capacity, suggesting that liver fibrosis or cirrhosis might indeed compromise the tolerance function of LSECs (19). On the other hand, dedifferentiated LSECs have been reported to exhibit reduced immunogenicity, marked by reduced expression of various scavenger receptors (20), reduced scavenger function (21) and induction of T cell exhaustion (22), suggesting that fibrosis might not compromise LSEC tolerance. Clarification of that issue is of particular clinical and translational importance. As LSECs are ‘gatekeepers of hepatic immunity’ (2) that recruit and regulate the composition of hepatic immune cell populations in all forms of acute or chronic hepatitis, maintenance or loss of their tolerance function is relevant for the development of liver diseases. Moreover, an impaired tolerance function of LSECs can contribute to systemic inflammation and the pathogenesis of cirrhosis-associated immune dysfunction (23). Furthermore, if the scavenger and tolerance functions of LSECs were compromised in liver fibrosis, it would interfere with nanomedicine applications that exploit LSEC functions (1).

Single-cell sequencing data comparing normal and cirrhotic human liver-derived non-parenchymal cells indicated a significant reduction of endothelial cells with a normal LSEC signature in cirrhotic livers (24), supporting the notion that LSEC tolerance might be impaired in cirrhosis. However, single-cell RNA sequencing from cirrhotic livers is problematic, since fibrosis hinders the dissociation of singlet cells, potentially producing biased cell compositions and isolation stress-induced alterations (25, 26). In order to avoid such bias, Su et al. performed a single-cell sequencing study in a mouse model of cirrhosis using Cdh5-CRE driven reporter activity to sort singlet LSECs, showing a reduced expression of scavenger receptors, but nonetheless a general preservation of LSEC identity (20). As yet it is not clear whether the observed reduced expression of scavenger receptors translates into dysfunctional scavenging and tolerance induction. Therefore, we now performed functional experiments using two mouse models of liver fibrosis, carbon tetrachloride (CCl_4_)-induced fibrosis (27) or spontaneous fibrosis in *Mdr2*-knockout mice (28), administering antigen-loaded nanoparticles in the context of extrahepatic and hepatic autoimmune disease models. Our findings indicate a robust maintenance of scavenger and immune tolerance functions by LSECs in liver fibrosis. Moreover, as fibrosis-induced transcriptional changes were similar between murine and human LSECs, our functional findings in mice seem to be transferable to humans.

## 2 Methods

### 2.1 Mice and fibrosis models

Inbred C57BL/6J, C57BL/6-Tg(TcraTcrb)1100Mjb/J (OT-1), B6.Cg-Abcb4^tm1Bor^/J (*Mdr2*-knockout) (28), K14-OVAp (10), K14-OVAp-*MDR2*KO (29) and C57BL/6-Tg(Tcra2D2,Tcrb2D2)1Kuch/J (2D2) (30) mice were bred and maintained in the animal facility of the University Medical Center Hamburg-Eppendorf under specific pathogen-free conditions. Liver fibrosis was induced by twice weekly intraperitoneal administration of CCl_4_ (Merck, 1:4 in corn oil (Sigma), 0.5ml/kg) over 4 weeks in 6–12 weeks old mice of both sexes; control mice received corn oil without CCl_4_. Alternatively, spontaneous liver fibrosis was assessed in 9–27 weeks old *Mdr2*-knockout mice. Representative findings confirming histological fibrosis induction, an increase of Sirius Red-stained areas, elevated serum ALT as marker of liver injury, and induction of fibrosis-associated gene expression by quantitative PCR of *Col1a1*, *Col3a1* and *Acta2* are shown in **Supplementary Figure 1A** (*Mdr2*-knockout) and **Supplementary Figure 1B** (CCl_4_). In all animal experiments, clinical and behavioral humane endpoints were applied to reduce pain and distress. All animal experiments were carried out in accordance with the principles of the Basel Declaration, the European Directive 2010/63/EU and FELASA recommendations, and had been approved by the animal experimentation review board of the State of Hamburg.

### 2.2 Cell isolation

Non-parenchymal liver cells were isolated as described before (4). Briefly, mouse livers were perfused with collagenase buffer (1 mg/ml collagenase type 2 (Worthington) in Gey’s balanced salt solution), dissected mechanically and further digested in collagenase buffer for 25 min at 37°C. Debris and hepatocytes were sedimented and non-parenchymal cells were recovered by gradient centrifugation (17% Optiprep). For isolation of LSECs, cells were further separated by CD146+ MACS according to manufacturer’s instructions. LSECs were pre-cultured overnight in collagenized 96-well cell culture plates in in Iscove’s Modified Dulbecco’s Medium (Sigma) supplemented with 10% FCS (PAA Laboratories) and 1% Pen/Strep (Gibco Life Technologies) at a density of 1.5x10^5^ cells per well before stimulation or co-cultures with T cells.

### 2.3 Analysis of immune molecule expression

**Figure 1:**
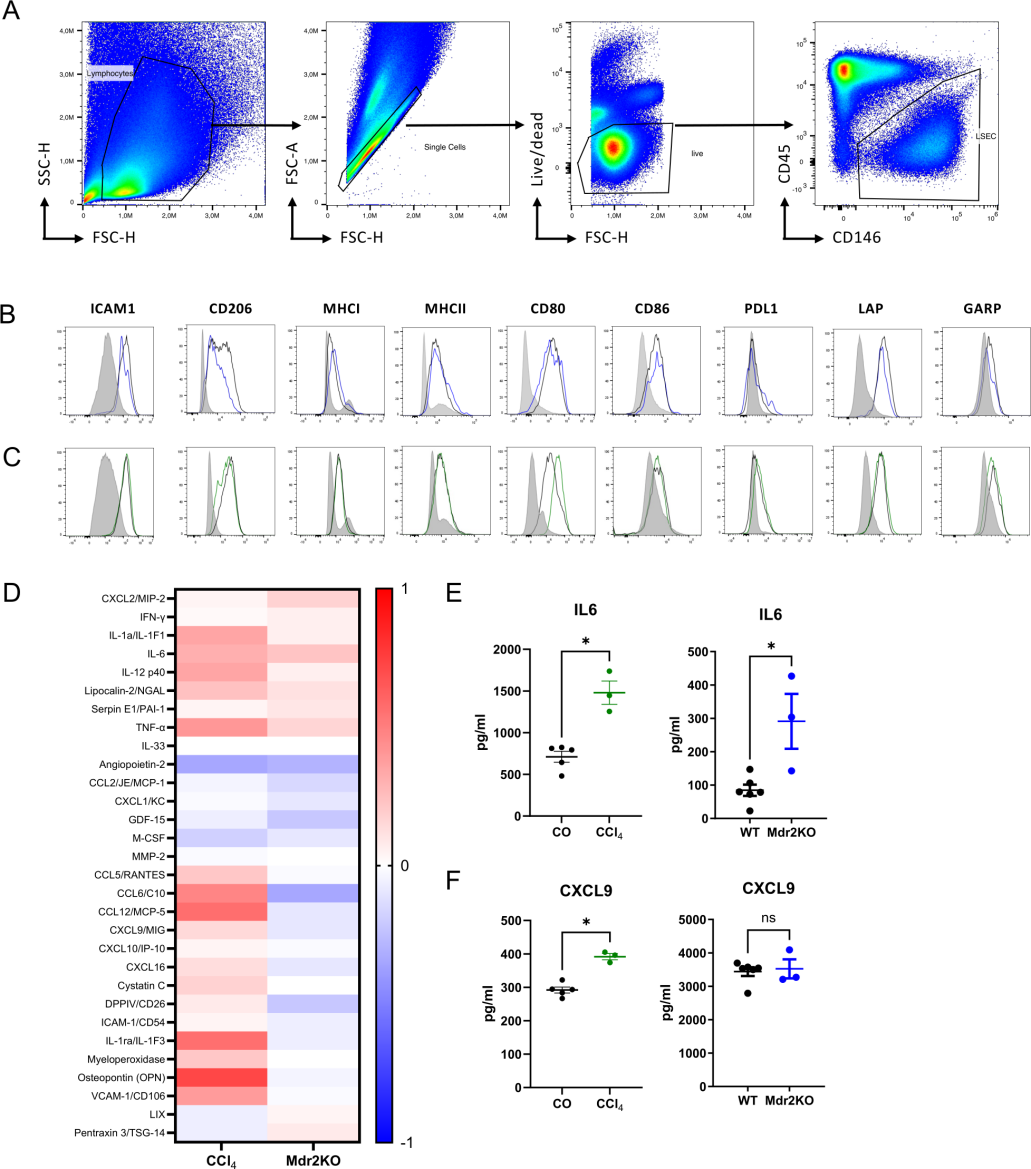
Expression of immune molecules and response to inflammatory stimulation by liver sinusoidal endothelial cells (LSECs). **(A)** Flow cytometry gating strategy to identify LSECs in liver non-parenchymal cells according to size and granularity, as living singlets and based on CD45 and CD146 expression. **(B, C)** Flow cytometric analysis of selected proteins associated with scavenging, adhesion, or tolerance by LSECs isolated from livers of fibrotic *Mdr2*-knockout mice (blue lines and dots) compared to non-fibrotic control LSECs (black lines and dots) **(B)**, or fibrotic livers of carbon tetrachloride-treated mice (green lines and dots) compared to non-fibrotic control LSECs (black lines and dots) **(C)**. Non-LSEC cells (grey) were used as benchmark. Shown are representative histograms for indicated molecules. **(D)** LSECs from non-fibrotic or fibrotic livers were isolated by magnetic separation with CD146 beads and taken into culture for 24h, followed by another 24h of stimulation with LPS (10μg/ml) and IFNγ (10ng/ml). The supernatants were semi-quantitatively analyzed with a Proteome Profiler (Mouse XL Cytokine Array). Fibrosis-induced changes in *Mdr2*-knockout fibrosis and carbon tetrachloride fibrosis are shown in a heat map as log (10) fold change after normalizing the signal intensities to their respective controls. **(E)** Exemplary validation of IL-6 response by LPS-stimulated LSECs, confirming consistent up-regulation in both fibrosis models. **(F)** Exemplary validation of CXCL9 response by IFNγ-stimulated LSECs, confirming up-regulation only in the carbon tetrachloride-induced fibrosis model. ns = non-significant; *P < 0.05.

Non-parenchymal cells were stained for their surface expression of ICAM1, CD206, MHCI, MHCII, CD80, CD86, LAP and GARP; antibodies are listed in **Table 1**. LSECs were identified based on CD45 and CD146 expression in living singlets as shown in **Figure 1A**, while non-LSEC were used as benchmark comparison.

**Table 1:** Antibodies used for flow cytometry (1:100 dilution).

Antigen	Clone	Conjugate	Catalog Nr	Company
CD4	RM4-5	AF532	58-0042-82	eBioscience
CD8a	53-6.7	APC/Fire 750	100766	Biolegend
CD44	IM7	PE/Dazzle 594	103056	Biolegend
CD45	30-F11	BV711	103147	Biolegend
CD45	30-F11	AF700	103128	Biolegend
CD45	30-F11	BV650	103151	Biolegend
CD45	30-F11	BV750	103157	Biolegend
CD45.1	A20	AF700	110724	Biolegend
CD49b	HMa2	PerCP-Cy5.5	103520	Biolegend
CD54 (ICAM1)	3E2	PE	553253	BD
CD62L	MEL-14	PE/Cy7	104418	Biolegend
CD80	16-10A1	AF594	104754	Biolegend
CD86	GL-1	BV785	105043	Biolegend
CD146	ME-9F1	PE/Cy7	134713	Biolegend
CD206	CO68C2	BV421	141717	Biolegend
CD223 (LAG3)	C9B7W	APC	125210	Biolegend
CD274 (PD-L1)	MIH5	BUV615	752339	BD
CD279 (PD1)	29F.1A12	APC/Cy7	135224	Biolegend
CD366 (TIM3)	RMT3-23	APC	119706	Biolegend
FOXP3	FJK-16s	FITC	11-5773-82	Invitrogen
GARP	YGIC86	eFluor450	48-9891-80	eBioscience
GrzmB	GB11	FITC	515403	Biolegend
H-2Kb	AF6-88.5.5.3	APC	17-5958-80	eBioscience
H-2Kb/SIINFEKL	25-D1.16	APC	141606	Biolegend
I-A[b]	AF6-120.1	BV421	562928	BD
IL10	JES5-16E3	PE/Dazzle594	505033	Biolegend
LAP	TW7-16B4	FITC	141414	Biolegend
TCR Va3.2	RR3-16	FITC	135403	Biolegend
TCR Vb11	RR3-15	PE	139003	Biolegend

### 2.4 LSEC stimulation

LSECs were stimulated with LPS (10 µg/ml, Merck) and IFNγ (10 ng/ml, Biolegend) for 24 h before collection of supernatants. Using these, Proteome Profiler Mouse XL Cytokine Array (R&D Systems) and ELISAs for IL-6 and CXCL9 (R&D Systems) were performed according to manufacturer’s instructions (**Table 2**). Intensity density of blots was determined using Fiji software with DotBlot Analysis.ijm.

**Table 2:** kits used for this manuscript.

Kit	Catalog Nr	Company
Elisa Mouse CXCL9/MIG DuoSet	DY492-05	R&D Systems
Elisa Mouse IL-6 DuoSet	DY406-05	R&D Systems
Foxp3 Staining Buffer Set	00-5523-00	Applied Biosystems
Proteome Profiler Mouse XL Cytokine Array	ARY028	R&D Systems

### 2.5 Treg conversion

Non-Treg CD4 T cells were isolated from spleens of 2D2 mice by MACS sorting of CD4+CD25- cells and splenic DC were isolated by MACS sorting of CD11c+ cells according to manufacturer’s instructions. Treg conversion assays were performed in serum-free Panserin medium (PAN-Biotech) by stimulation of non-Treg CD25- CD4+ T cells (5x10^5^/well) on LSECs (1.5x10^5^/well) or splenic DC (5x10^4^/well) with soluble anti-CD3 antibody (2 μg/ml, BD Biosciences) or MOG (MEVGWYRSPFSRVVHLYRNGK, Panatecs, Germany) peptide (2 μg/ml), both in the presence of 2 ng/ml recombinant hTGFβ1(Miltenyi). Intracellular staining of FoxP3 was performed by using FoxP3 Staining Kit (Invitrogen) according to manufacturer’s instructions (**Table 2**). The percentage of Tregs (defined as CD4+ FoxP3+ cells) was analyzed on day 4 of co-culture using flow cytometric measurements.

### 2.6 Peptide-conjugated nanoparticles

Oleic acid-stabilized superparamagnetic iron oxide nanoparticles encapsulated in an amphiphilic polymer (poly(maleic anhydride-alt-1-octadecene)) were coupled to peptides as described previously (11). Nanoparticles were provided by Topas Therapeutics in a D-Mannitol (5% w/v), L-Lactic Acid (6 mM), and TRIS (5 mM) buffer.

### 2.7 Liver-specific cellular distribution of nanoparticles

Liver-specific cellular distribution of nanoparticles was analyzed as described before (11). Briefly, mice were intravenously injected with Cy5-labelled nanoparticles and sacrificed after 5 min. NPC were isolated, and analyzed by flow cytometry identifying LSECs as CD45- CD146+ as shown **Figure 1A** for percentage of Cy5^+^ scavenging LSECs in comparison to non-LSECs, and their Cy5-MFI.

### 2.8 Cross-presentation of OVA

Cross-presentation of ovalbumin peptide (OVA) was assessed as described before (10). Briefly, isolated LSECs were incubated in Iscove’s Modified Dulbecco’s Medium 10% FCS 1% Pen/Strep with nanoparticles conjugated to OVA peptide (10 µM and 100 µM) or unconjugated nanoparticles for 16 h at 37 °C, 5% CO_2._ Cells were washed and stained with antibodies recognizing OVA peptide on H2-Kb, CD146 and CD45 and analyzed via flow cytometry as seen in **Figure 1A** for Cy5-MFI.

### 2.9 Flow cytometry

Measurements were performed at a Cytek Aurora 5-Laser Flow Cytometer and analyzed with FlowJo v10 using single stains for unmixing and compensation. Dead cells were excluded by incubation with Zombie Aqua or PacO (1:1000 dilution) for 15min in the dark. Cells were stained extracellularly for 20min at 4 °C in the dark for most markers, while staining LAG3 required staining for 15min at 37 °C and then 15min at room temperature in the dark. Antibodies were used in a 1:100 dilution, as listed in **Table 1**. Fixation and intracellular staining were performed by using FoxP3 Staining Kit (Invitrogen). Positive populations were gated in comparison to negative populations within the analyzed sample, overlays in histograms are shown as normalized counts and MFI was defined as median fluorescence intensity.

### 2.10 Experimental autoimmune encephalomyelitis

EAE was induced as described before (9). Briefly, mice were injected subcutaneously at the base of the tail with an emulsion of 100 µg MOG peptide and CFA containing 4 mg/ml heat-killed *Mycobacterium tuberculosis* strain H37RA. 200 ng of pertussis toxin was injected i.p. in 200 µl of PBS on the day of immunization and two days later. MOG peptide-conjugated nanoparticles (20 nmol) or unconjugated control nanoparticles were i.v. injected in the tail vein one day prior to immunization. Disease severity was monitored daily for 30 days post immunization. Clinical symptoms were scored as follows: 0, no detectable signs of EAE; 1, tail atony; 2, partial hind limb paralysis; 3, complete hind limb paralysis; 4, fore limb and hind limb paralysis; 5; moribund. For ethical reasons, mice were sacrificed upon approaching score 4. For immunophenotyping of T cells in EAE, 5x10^6^ splenic CD4+ T cells from MOG-specific 2D2 were transferred intravenously on day 1 after immunization, and NPC were isolated from liver tissue on day 7 after immunization. Antigen-specific CD4 T cells were defined as TCRVα3.2+ TCRVβ11+.

### 2.11 Autoimmune cholangitis

Autoimmune cholangitis was induced by i.v. transfer of CD8+ OT-I T cells into K14OVAp mice as described previously (10). OVA-conjugated nanoparticles (20 nmol) or unconjugated control nanoparticles were injected one day prior to OT-I transfer. Disease severity was scored daily by assigning 0–2 points (0: unaffected, 1: moderate impairment, 2: severe impairment) to body weight, appearance of fur and eyes, mobility and bearing and summed. Five to six days after OT-I T cell transfer, immunophenotyping of hepatic infiltrates and splenic cells was performed. Transferred autoreactive CD8 T cells were identified by their expression of CD45.1.

### 2.12 Single-cell sequencing analyses

We used public mouse scRNA sequencing data from Su et al. (GSE147581) (20) and the pre-processed scRNA sequencing data from Andrews et al. (31). For the mouse data, we followed closely the previously described methods to reproduce the clusters reported by Su et al. Briefly, cells with a mitochondrial gene expression percentage > 20%, fewer than 200 genes or eGFP-mTmG count <= 5 were removed. The remaining expression data was normalized using Seurat’s (version 5.3.0) LogNormalize approach (32). After identifying highly variable genes and running PCA on the scaled data, we performed Seurat’s CCA data integration between both mouse datasets. We used the first 30 components of the corrected embedding for the neighborhood graph construction and applied the Leiden algorithm with a resolution of 0.5 to recreate a similar amount of EC clusters. We annotated the resulting cluster according to their overlap with cluster markers reported by Su et al. For further analysis, we merged the clusters EC2, 3 and 4 as LSEC and cluster EC1 and 5 as endothelial cell (EC). From the human scRNA sequencing data we merged the endothelial cell of periportal hepatic sinusoid and endothelial cell of pericentral hepatic sinusoid clusters as LSEC. For both data sets, we performed differential gene expression analysis between fibrosis groups within the LSEC and EC populations using Libra (version 1.0.0) (33). For the human data, we used pseudo-bulk comparisons with edgeR and, due to insufficient sample numbers, we used single cell comparisons with the Wilcoxon Rank Sum test with FDR correction for the mouse data. Over-representation analysis was performed with clusterProfiler (version 4.16.0) (34) and gene set databases provided by MsigDB (35). The threshold for statistical significance was an FDR below 0.05. All analyses were performed in R (version 4.5.1).

### 2.13 Statistics

Experiments were repeated at least twice, apart from targeting experiments, which were performed once. Statistical analysis of two groups was performed using the Mann-Whitney U test. Comparison of three or more groups was conducted with the Kruskal-Wallis-Test and Dunns’s *post-hoc* test. A p value <0.05 was considered statistically significant and significances were marked as follows: *p<0.05, **p<0.01, ***p<0.001, ****p<0.0001. All data were analysed with GraphPad Prism 10.

## 3 Results

To approach the scavenger and tolerance function of LSECs in fibrosis, we employed two different mouse models of liver fibrosis, the model of CCl_4_-induced fibrosis that had also been used by Su et al. (20), and spontaneous fibrosis in *Mdr2*-knockout mice (28). First, we used these mouse models to determine the presence of representative molecules with scavenger or tolerance functions of LSECs in fibrosis. To that end, we isolated non-parenchymal liver cells from wildtype control mice or from fibrotic livers of *Mdr2*-knockout or CCl_4_-treated mice and *ex vivo* analyzed these molecules by flow cytometry; LSECs were gated as CD146+CD45- cells and the CD146- non-LSECs were used as benchmark and internal control (**Figure 1A**). Some molecules, including ICAM1, CD206, PD-L1, LAP and GARP showed slightly reduced median fluorescence intensities (MFI) in LSECs from fibrotic *Mdr2*-knockout mice compared to LSECs from non-fibrotic control mice (**Figure 1B**); however, these molecules were not altered in CCl_4_-treated mice, which showed upregulated expression of CD80 instead (**Figure 1C**). Although some of these changes in fibrotic LSECs were significant (**Supplementary Figure 2**), the expression levels of these molecules clearly remained higher and distinct from that of non-LSECs, indicating that LSECs largely maintained a characteristic protein profile in liver fibrosis with respect to the selected scavenger, adhesion or tolerance molecules.

Since LSECs isolated from fibrotic livers were reported to show an increased production of pro-inflammatory mediators (19), we next isolated LSECs from fibrotic mice of both models or from respective non-fibrotic control mice by CD146-based magnetic separation, and stimulated these cells *in vitro* with LPS and IFNγ to detect potential pro-inflammatory activation. After 24 hours, the supernatants were semi-quantitatively analyzed with a Proteome Profiler Kit (**Supplementary Figures 3A, B**), and the respective signal intensities of the two fibrosis models were normalized to their respective controls. **Figure 1D** shows a heat map of the detectable fibrosis-induced changes of proteins expressed above a minimal threshold in both fibrosis models as log (10) fold change relative to respective controls. Some chemokines and cytokines, such as IL-6 or TNF were consistently up-regulated in both fibrosis models, whereas others, such as angiopoietin-2, CCL2 or M-CSF were consistently down-regulated. Yet other factors, such as CXCL9 or CCR5 were not consistently regulated in the two fibrosis models. To confirm the validity of the Proteome Profiler, we analyzed the supernatants also by ELISA for IL-6 (**Figure 1E**) or CXCL9 (**Figure 1F**), as examples for factors regulated in response to LPS or IFNγ, respectively. The ELISA findings confirmed consistent induction of IL-6 by LPS in both models, and induction of CXCL9 by IFNγ only in the CCl_4_ model. Together, these findings indicated that fibrosis induced a partially model-dependent up- or down-regulation of discrete inflammatory mediators in LSECs, but not a general pro-inflammatory transition of LSECs.

**Figure 2:**
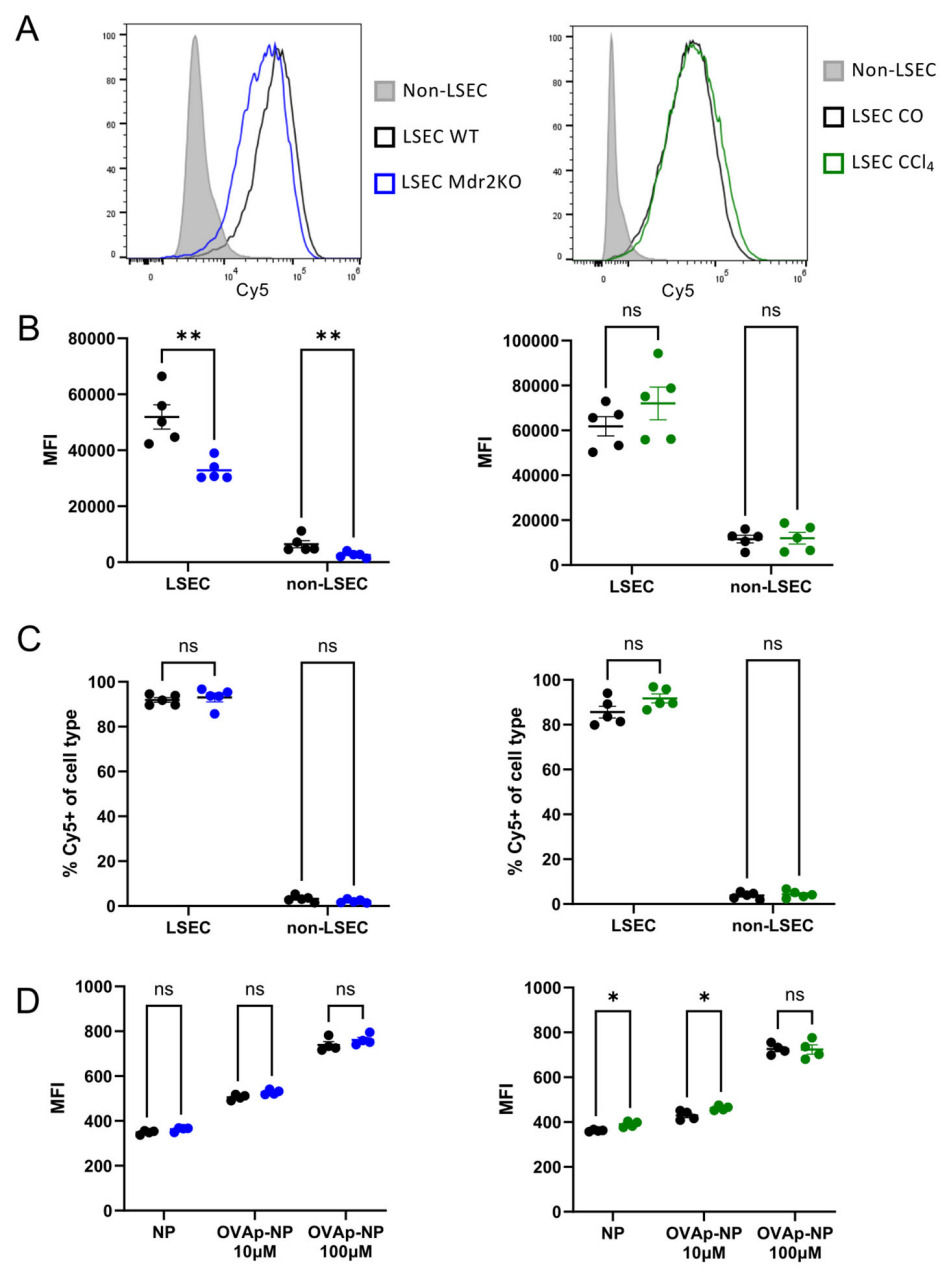
LSEC scavenger function. LSEC-targeting nanoparticles labelled with Cy5 were administered intravenously to fibrotic or non-fibrotic control mice, and nanoparticle uptake by liver non-parenchymal cells was assessed *ex vivo* with flow cytometry after 5 min. **(A)** Shown in the left panel is a representative histogram of incorporated Cy5 fluorescence of LSECs from wildtype (black line) or *Mdr2*-knockout mice (blue line) and non-LSECs (grey) as benchmark. Shown in the right panel is a representative histogram of incorporated Cy5 fluorescence of LSECs from corn oil (CO)-treated control mice (black line) or CCl_4_-treated mice (green line) and non-LSECs (grey) as benchmark. **(B)** Median fluorescence intensity (MFI) of LSECs and non-LSECs from control (black dots) or *Mdr2*-knockout mice (blue dots) (left panel), and of LSECs from corn oil-treated (black dots) or CCl_4_-treated mice (green dots (right panel). **(C)** Percentage of Cy5^+^ scavenging LSECs in comparison to non-LSECs, each from 5 control (black dots) or 5 *Mdr2*-knockout mice (blue dots) (left panel), and from 5 corn oil-treated (black dots) or 5 CCl_4_-treated mice (green dots (right panel) (n=5). **(D)** The capacity of LSECs from non-fibrotic or fibrotic livers to cross-present ingested nanoparticle-bound peptides was assessed taking advantage of an antibody that recognizes an ovalbumin-derived peptide (OVA) within the context of H2-K^b^ MHC-I molecules. The detection of K^b^-bound ovalbumin peptide 16 hours after administration of nanoparticle-bound ovalbumin peptide in the *Mdr2*-knockout (blue dots, left) and the CCl_4_-induced model (green dots, right) is shown in comparison to their respective non-fibrotic controls (black dots). ns = non-significant; *P < 0.05; **P < 0.01.

Next, to investigate the scavenger activity of LSECs, we administered LSEC-targeting nanoparticles labelled with Cy5 (11) to mice with or without liver fibrosis and, after 5 min, assessed their uptake by liver non-parenchymal cells using flow cytometry *ex vivo*, applying the gating scheme shown in **Figure 1A**. LSECs were the most effective scavenger cells for these nanoparticles in all conditions, indicated by marked elevation of Cy5^+^ fluorescence intensity compared to non-LSECs (grey), both in the *Mdr2*-knockout and the CCl_4_-induced model. Representative histograms of Cy5 fluorescence in LSECs in the *Mdr2*-knockout and the CCl_4_-induced model as compared to non-LSECs are shown in **Figure 2A**. Median Cy5 fluorescence intensity (MFI) (**Figure 2B**) indicated that in *Mdr2*-knockout but not in CCl_4_-induced fibrosis, nanoparticle uptake was significantly reduced, but nonetheless clearly more efficient than that of non-LSECs. Thus, although liver fibrosis may slightly reduce nanoparticle uptake kinetics, it did not extensively compromise LSEC scavenger function. Moreover, about 90% of all LSECs had taken up Cy5^+^ nanoparticles, and the frequency of scavenging LSECs was not significantly changed in the *Mdr2*-knockout and the CCl_4_-induced model (**Figure 2C**). We then compared the capacity of LSECs from non-fibrotic and fibrotic livers to cross-present ingested nanoparticle-bound peptides, taking advantage of an antibody that recognizes an ovalbumin-derived peptide (OVA) within the context of H2-K^b^ MHC-I molecules (10). As shown in **Figure 2D**, the differences in the capacity of fibrotic or non-fibrotic LSECs to cross-present nanoparticle-bound peptide were negligible, indicated by the detection of K^b^-bound ovalbumin peptide 16 hours after administration of nanoparticle-bound ovalbumin peptide, both in the *Mdr2*-knockout and the CCl_4_-induced model. Thus, antigen uptake, processing and cross-presentation by LSECs was not impaired by liver fibrosis.

**Figure 3:**
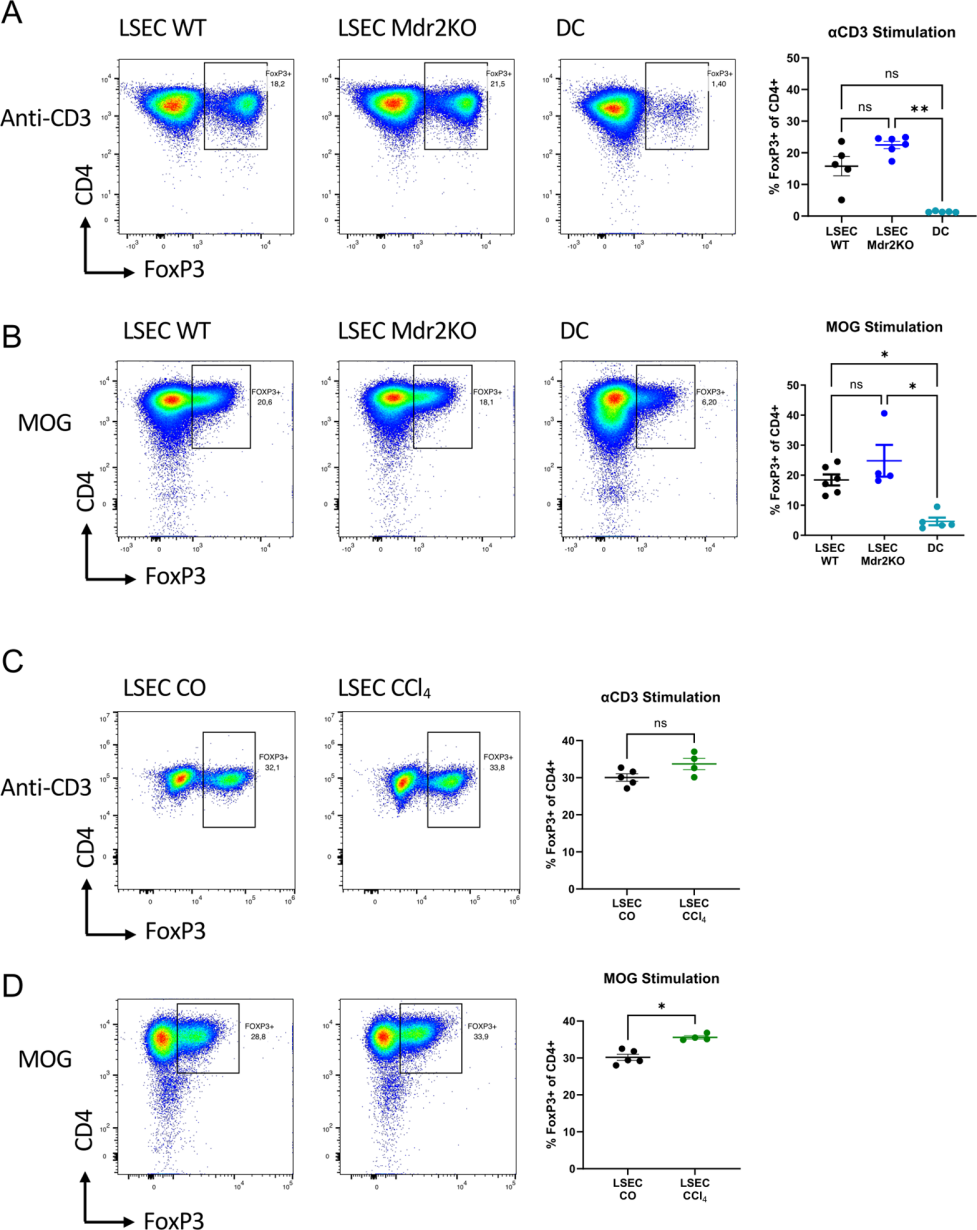
Induction of regulatory T cells by LSECs. MOG-specific CD4+ CD25- T cells from 2D2 mice were co-cultured with LSECs from non-fibrotic control or fibrotic livers in the presence of 2ng/ml TGFβ and stimulated with soluble anti-CD3 antibody or MOG peptide; as indicated, T cell stimulation on splenic dendritic cells served as benchmark. After 4 days Treg-induction was assessed by staining for Foxp3 in flow cytometry. Shown are representative dot plots for anti-CD3 stimulation **(A)** and MOG stimulation **(B)** comparing LSECs from *Mdr2*-knockout or control livers and dendritic cells, as well as statistical results (right). **(C, D)** Treg induction by LSECs from non-fibrotic livers of corn oil-treated mice or from fibrotic livers of CCl_4_-treated mice; shown are representative dot plots for anti-CD3 stimulation **(C)** and MOG stimulation **(D)** and statistical results (right). ns. non-significant; *P < 0.05; **P < 0.01.

It has been reported that LSECs from fibrotic livers were compromised in their capacity to induce regulatory T cells (Treg) (19). However, the significance of these experiments can be questioned, as they were performed without stimulation, and in conventional media supplemented with serum, containing highly variable TGFβ activity that is critical for peripheral Treg induction (36). Therefore, we here assessed Treg induction by LSECs from fibrotic or non-fibrotic livers in co-culture with CD25-negative non-Treg CD4 T cells with serum-free medium in the presence of a defined dose of 2 ng/ml TGFβ, and with antigen-specific or anti-CD3 T cell stimulation. LSECs from *Mdr2*-knockout mice were highly effective inducers of Tregs when stimulating T cells non-specifically with soluble anti-CD3 antibody (**Figure 3A**), or specifically with MOG peptide (**Figure 3B**). Of note, both in fibrotic *Mdr2*-knockout or wildtype mice, Treg induction by LSECs was considerably more effective than that of splenic dendritic cells, which were used as benchmark (**Figures 3A, B**). Likewise, we also found no difference in Treg induction between LSECs from fibrotic CCl_4_-treated mice or control mice, neither following non-specific anti-CD3 stimulation (**Figure 3C**) nor specific stimulation with MOG peptide (**Figure 3D**). These findings demonstrated that liver fibrosis did not compromise the Treg-inducing capacity of LSECs.

**Figure 4:**
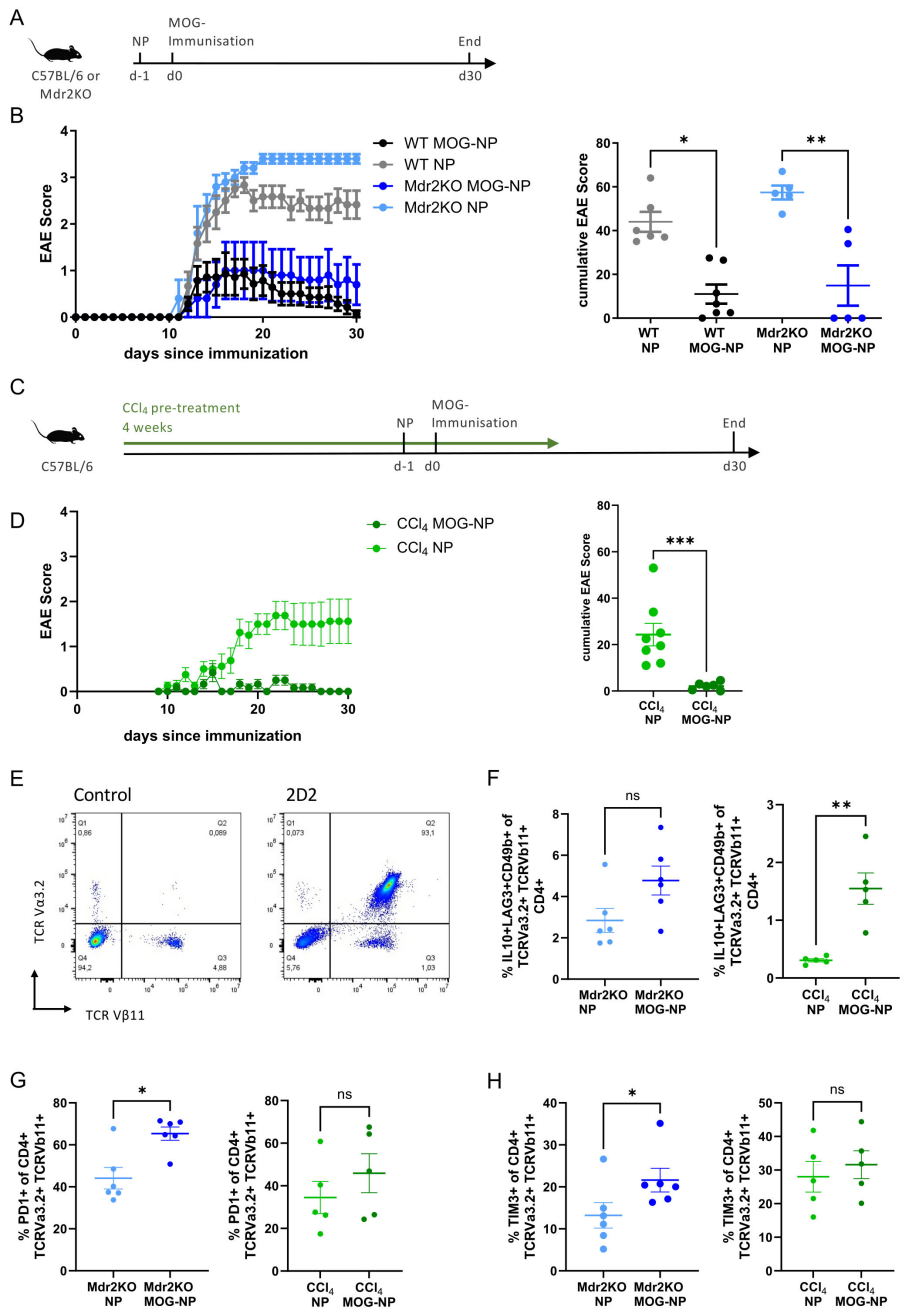
Antigen-specific tolerance induction and protection from CD4 T cell-mediated autoimmune disease using LSEC-targeting nanoparticles. **(A)** Experimental setup testing protection from MOG-induced experimental autoimmune encephalomyelitis (EAE) in fibrotic *Mdr2*-knockout or non-fibrotic C57BL/6 control mice. LSEC-targeting nanoparticles conjugated with MOG peptides (MOG-NP) or unconjugated control nanoparticles (NP) were administered intravenously, one day later followed by EAE induction through immunization to MOG; EAE symptoms were then scored for 30 days. **(B)** Disease course was plotted by a clinical EAE score in *Mdr2*-knockout or control mice, comparing MOG-NP and NP (left). Cumulative disease score for each animal over the 30-day observation period is shown on the right. **(C)** Experimental setup testing protection from MOG-induced EAE in fibrotic or non-fibrotic C57BL/6 mice; fibrosis was induced by administration of carbon tetrachloride over four weeks. LSEC-targeting nanoparticles conjugated with MOG peptides (MOG-NP) or unconjugated control nanoparticles (NP) were then administered intravenously, and one day later, EAE was induced by immunization to MOG, followed by three additional carbon tetrachloride administrations in the first one and a half weeks after MOG immunization; EAE symptoms were scored for 30 days following MOG immunization. **(D)** Disease course was plotted by a clinical EAE score in fibrotic vs. non-fibrotic mice, comparing MOG-NP and free NP treatment (left). Cumulative disease score for each animal over the 30-day observation period is shown on the right. E) MOG-specific 2D2 T cells (5x10^6^ per mouse) were transferred 1 day after EAE induction, retrieved at day 7 and analyzed by flow cytometry. By staining for TCR Va3.2 and TCR Vb11 chains (right), transferred 2D2 cells could be identified against the background without T cell transfer (left). **(F)** Increase of MOG-specific Tr1-like cells following MOG-NP treatment in livers of Mdr2KO mice (left) or CCl4-treated mice (right). Tr1-like cells were defined as CD49b+ LAG3+ IL10+ TCRVa3.2+ TCRVb11+ CD4+ T cells. **(G)** MOG-NP treatment induced an increase in PD-1 expression on MOG-specific CD4 T cells, both in livers of Mdr2KO mice (left) or CCl4-treated mice (right). **(H)** MOG-NP treatment induced an increase in TIM3 expression on MOG-specific CD4 T cells, both in livers of Mdr2KO mice (left) or CCl4-treated mice (right). ns = non-significant; * P < 0.05; ** P < 0.01; *** P < 0.001.

We then assessed the efficacy of antigen-specific tolerance induction in CD4 T cells *in vivo* utilizing LSEC-targeting nanoparticles in mice with liver fibrosis. First, we administered nanoparticles conjugated with MOG peptides or unconjugated control nanoparticles to wildtype or *Mdr2*-knockout mice the day before induction of EAE by immunization to MOG peptide (**Figure 4A**). Whereas peptide-free control nanoparticles could not protect neither wildtype nor *Mdr2*-knockout mice from CD4 T cell-driven disease development, MOG peptide-conjugated nanoparticles were similarly protective from disease development both in wildtype and *Mdr2*-knockout mice, as seen by disease course (**Figures 4B**, left) and cumulative disease scores (right). These findings were confirmed in the model of CCl_4_-induced fibrosis (**Figure 4C**) in which administration of MOG-conjugated nanoparticles likewise provided disease protection, whereas unconjugated control nanoparticles could not protect from EAE development (**Figure 4D**). To assess potential T cell tolerance induction against MOG, we varied the experiment by performing adoptive transfer of 5x10^6^ MOG-specific 2D2 CD4 T cells one day after EAE induction, which we then retrieved and analyzed based on their expression of the TCR Va3.2 und TCR Vb11 chains, defining a distinct 2D2-enriched population against very low background (**Figure 4E**). Following MOG delivery to LSECs, we found an increase in MOG-specific Tr1-like cells, which were defined using established markers as CD49b+ LAG3+ IL10+ TCRVa3.2+ TCRVb11+ CD4+ T cells (37); this increase was significant in the CCl_4_ model and by trend in *Mdr2*-knockout mice (**Figure 4F**). Moreover, we found increased expression of both PD-1+ (**Figure 4G**) or TIM3+ (**Figure 4H**) on MOG-specific TCRVa3.2+ TCRVb11+ CD4+ T cells, significant in *Mdr2*-knockout mice and by trend in the CCl_4_ model; expression of these markers has been shown before to be associated with liver-induced T cell tolerance (11, 38).

**Figure 5:**
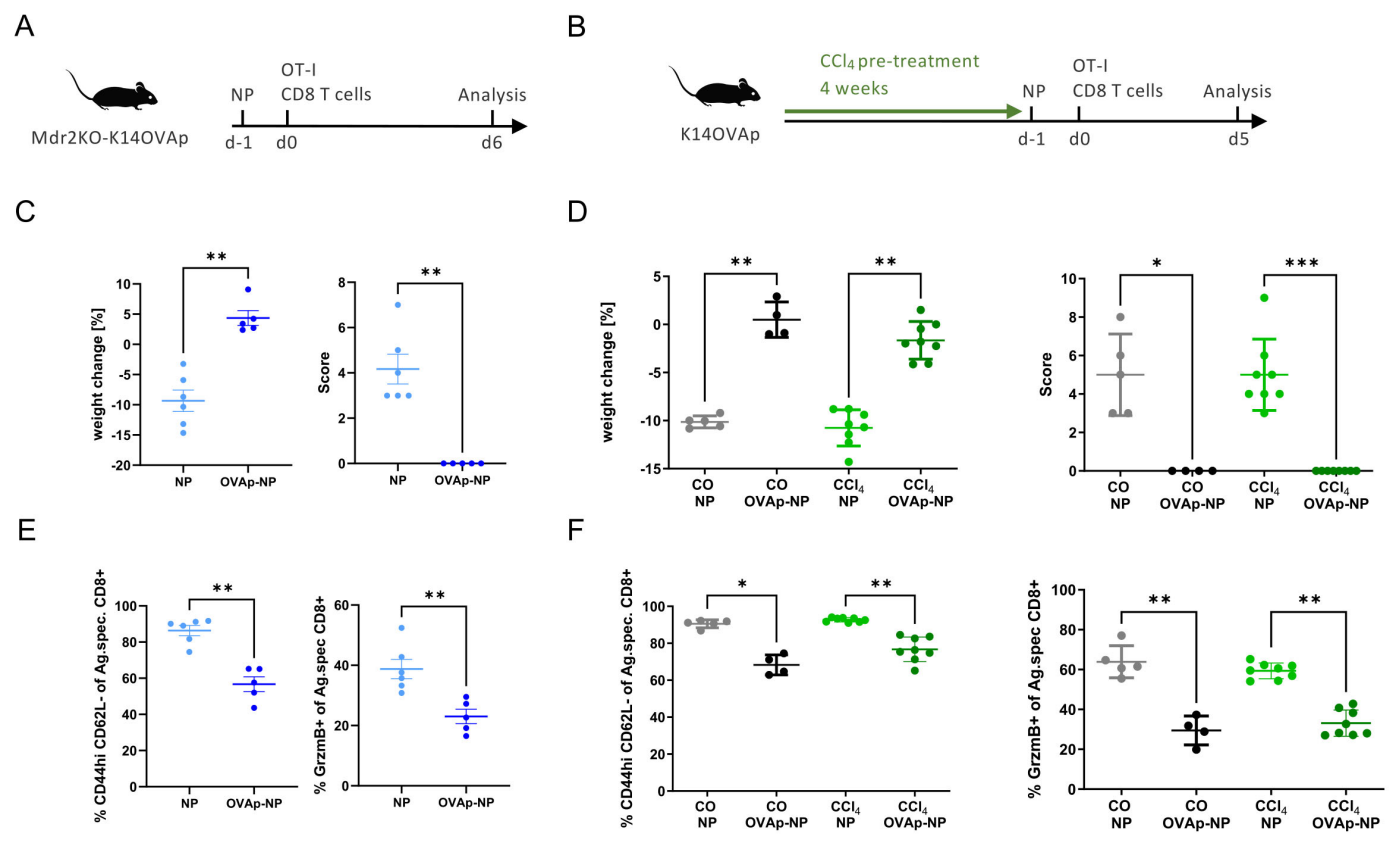
Antigen-specific tolerance induction and protection from CD8 T cell-mediated autoimmune disease using LSEC-targeting nanoparticles. **(A)** Experimental setup testing protection from OT1 CD8 T cell-induced cholangitis in fibrotic K14-OVAp mice on *Mdr2*-knockout background or non-fibrotic K14-OVAp control mice. LSEC-targeting nanoparticles conjugated with ovalbumin peptide (OVA-NP) or unconjugated control nanoparticles (NP) were administered intravenously, one day later followed by transfer of 2x10^5^ OT1 cells; disease development was observed for 6 days. **(B)** Experimental setup testing protection from OT1 CD8 T cell-induced cholangitis in fibrotic K14-OVAp mice after carbon tetrachloride treatment or non-fibrotic K14-OVAp control mice after corn oil treatment. LSEC-targeting nanoparticles conjugated with ovalbumin peptide (OVA-NP) or unconjugated control nanoparticles (NP) were administered intravenously, one day later followed by transfer of 2x10^5^ OT1 cells; disease development was observed for 5 days. **(C, D)** Disease course was assessed by weight change (left) and a clinical disease score (right) in Mdr2KO-K14OVAp mice **(C)** or in CCl_4_ treated K14-OVAp mice **(D)**. **(E, F)** Liver infiltrating cells were assessed by flow cytometry for proportion of OT1 effector cells, and their production of granzyme B, in Mdr2KO-K14OVAp mice **(E)** or in CCl_4_ treated K14-OVAp mice **(F)**. ns = non-significant; *P < 0.05; **P < 0.01; ***P < 0.001.

Furthermore, we studied LSEC-driven antigen-specific tolerance induction in CD8 T cells *in vivo* utilizing K14-OVAp mice expressing the ovalbumin-derived OVA peptide in cholangiocytes; these mice develop acute cholangitis following transfer of OT-I CD8 T cells that recognize the OVA peptide. Accordingly, LSEC-targeting nanoparticles conjugated with ovalbumin-derived OVA peptide were administered to K14-OVAp mice on *Mdr2*-knockout background, in comparison to unconjugated control nanoparticles (**Figure 5A**). Alternatively, a similar procedure was applied in the model of CCl_4_-induced liver fibrosis (**Figure 5B**). As expected, unconjugated nanoparticles could not prevent disease induction, assessed by weight loss and a symptomatic disease score; in contrast, OVA peptide-conjugated nanoparticles completely prevented weight loss and disease symptoms in the *Mdr2*-knockout model (**Figure 5C**) or in CCl_4_-induced fibrosis (**Figure 5D**). Protection was associated with significant reduction of liver-infiltrating antigen-specific OT-1 T cells with effector memory phenotype and granzyme B production, both in the *Mdr2*-knockout model (**Figure 5E**) or in CCl_4_-induced fibrosis (**Figure 5F**). Together, these findings indicated that in liver fibrosis, LSECs maintain their ability to induce antigen-specific tolerance in CD4 and CD8 T cells and facilitate protection from autoimmune disease, as effectively as under homeostatic conditions.

**Figure 6:**
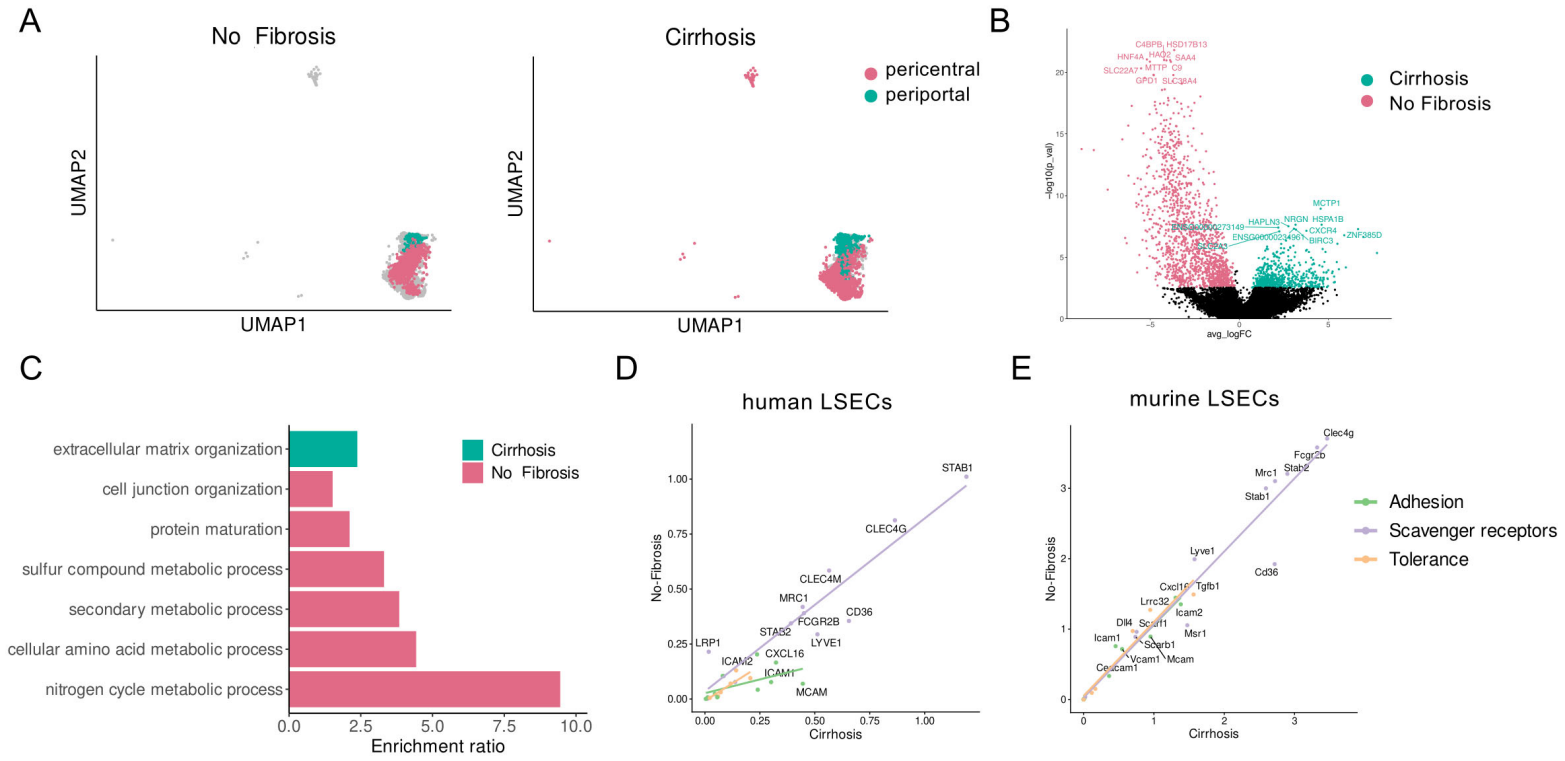
Single-cell RNA sequencing analysis of human and mouse liver sinusoidal endothelial cells (LSECs) in liver fibrosis. Published single-cell RNA sequencing data comparing non-fibrotic and cirrhotic human (29) and murine livers (19) were re-analyzed for conservation of LSEC phenotype. **(A)** Visual comparison of the UMAP projections for the human LSECs revealed that there was a high congruence of the periportal and pericentral LSEC clusters in the two analyzed conditions (no fibrosis, cirrhosis) and both conditions contributed to both clusters. **(B)** Differentially expressed genes between all human LSEC clusters from non-fibrotic livers and those from cirrhotic livers are represented as volcano plots. **(C)** Gene set enrichment analysis comparing human LSECs from cirrhotic and non-fibrotic livers revealed only few overrepresented pathways, which were linked to fibrosis, but not with scavenging, cell adhesion or tolerance. **(D)** Scatter plot correlating mean expression levels in cirrhotic vs non-cirrhotic human livers of selected molecules associated with scavenging, cell adhesion and tolerance, showing only a marginal increase of molecules related to adhesion in cirrhosis, but not of molecules related to scavenging or tolerance. **(E)** Scatter plot correlating mean expression levels in cirrhotic vs non-cirrhotic murine livers of selected molecules associated with scavenging, cell adhesion and tolerance, showing only marginal down-regulation of these molecules in cirrhosis.

Finally, to assess the transferability to human liver fibrosis, we used a published pre-processed single-cell RNA sequencing dataset (31) to compare the transcriptional data of the endothelial cell subset from cirrhotic and non-cirrhotic human livers. Visual comparison of the UMAP projections for these cells revealed that there was a high congruence of the periportal and pericentral LSEC clusters in the two analyzed conditions (no fibrosis, cirrhosis) and both conditions contributed to both clusters (**Figure 6A**). We then analyzed the differentially expressed genes, collectively comparing all LSEC clusters from non-fibrotic livers with those of cirrhotic livers (**Figure 6B**), represented as volcano plots. An over-representation analysis using gene ontology (GO) gene sets revealed that only few pathways were significantly enriched in cirrhosis or non-cirrhosis (**Figure 6C**). LSECs from cirrhotic livers were significantly enriched in gene sets related to organization of extracellular matrix, marking the endpoint of LSEC dedifferentiation (13). LSECs from non-cirrhotic livers were mainly enriched in a few pathways related to cell junction, protein maturation and metabolic processes (**Figure 6C**). These findings suggested that the transcriptional adaptations of LSECs in cirrhosis seemed to be restricted to few pathways directly associated with fibrogenesis, but not with pathways that are linked to the scavenger or tolerance function of LSECs. To further test this assumption, we specifically analyzed the expression of genes for scavenger receptors (*CD36*, CLEC4G, *CLEC4M*, *FCGBR2B*, *LRP1*, *LYVE1*, *MRC1*, *MSR1*, *SCARB1*, *SCARF1*, *STAB1*, *STAB2*) (1), adhesion molecules (*AOC3*, *CD209*, *CEACAM1*, *CXCL16*, *ICAM1*, *ICAM2*, *ITGB2*, *MCAM*, *VCAM1*) (2), and tolerance-associated molecules, including *CD274* (3), *PDCD1LG2* (39), *DLL4* and *JAG1* (40), as well as *LRRC32* and *TGFB1* (4), and correlated their mean expression in non-cirrhotic vs. cirrhotic livers as scatter plot (**Figure 6D**). These findings indicated that scavenger receptors and tolerance molecules were nearly unchanged between the two conditions, whereas adhesion molecules seemed to be marginally enriched in LSECs from cirrhotic livers. Thus, this transcriptional data seemed to confirm that the scavenger and tolerance functions of LSECs are maintained also in human liver fibrosis. For comparison, we also processed and represented the expression data of murine LSECs derived from the single-cell sequencing dataset by Su et al. (20) in a similar way, shown in **Figure 6E**. Although all three molecule types were marginally reduced in cirrhotic livers, murine LSECs likewise seemed to largely maintain expression of these molecules, as human LSECs. Since we have demonstrated above that murine, LSEC conserved their scavenger and tolerance functions *in vivo*, these findings suggest that human LSECs likewise retain scavenging and tolerance functions in liver fibrosis.

## 4 Discussion

In this study, we have assessed the scavenger and tolerance function of LSECs in liver fibrosis. First, we analyzed *ex vivo* isolated LSECs that were derived from two mouse models of liver fibrosis, finding similar expression of selected proteins involved in scavenging, cell adhesion or immune tolerance as in cells from non-fibrotic control livers (**Figure 1**). Moreover, LSECs from fibrotic livers responded to LPS and IFNγ stimulation with up- or down-regulation of specific cytokines and chemokines, but not with developing a general pro-inflammatory phenotype (**Figure 1**). A previous study (19) had reported an increase in pro-inflammatory mediators secreted by LSECs from fibrotic livers. While these results were consistent with some of our findings, e.g. TNF, they were not consistent with others, e.g. CXCL1 and CCL2. This discrepancy is likely explained by the assay protocol. Connolly et al. analyzed supernatant from unstimulated LSEC 24h after their isolation. In contrast, in order to avoid isolation stress-induced LSEC activation, we rested the isolated LSECs for 24 hours prior to stimulation with LPS and IFNγ in defined concentrations before analyzing the supernatants after another 24h. Importantly, considering the range of up- and down-regulation of different mediators, our findings did not indicate a general pro-inflammatory maturation of LSECs in liver fibrosis.

To directly assess the scavenger function of LSECs, we used Cy5-labelled LSEC-targeting nanoparticles (11), finding that nanoparticle scavenging by LSECs was not compromised by liver fibrosis (**Figure 2**). Moreover, peptides that were conjugated to those nanoparticles were similarly cross-presented by LSECs, both in fibrotic and non-fibrotic livers (**Figure 2**). Furthermore, we did not find significant differences in the ability of LSECs to induce Tregs (**Figure 3**). This finding contrasts with reports of decreased Treg induction by LSECs from fibrotic livers (19, 41). However, these studies had used standard medium supplemented with serum, containing undefined and variable TGFβ activity, and, importantly, no T cell stimulation, which is not optimal for assessing peripheral Treg induction. In contrast, we applied serum-free medium with a defined dose of TGFβ, and performed antigen-specific or non-specific T cell stimulation (**Figure 3**). Thus, our findings reliably reflect the Treg-inducing capacity of LSECs, which we find unaltered in liver fibrosis, and considerably greater than that of dendritic cells. Together these findings indicated that the scavenger and tolerance function of LSECs was not compromised by liver fibrosis. In accordance with our findings, a recent report indicated that LSECs can inhibit CD8 T cell functions also in liver fibrosis (22), lending further support to the notion that fibrosis does not seem to impair the tolerance function of LSECs.

However, we also directly assessed LSEC-induced tolerance in CD4 T cell-driven EAE, using nanoparticles loaded with myelin peptides, and found that antigen-specific tolerance was induced by LSECs both in healthy and fibrotic livers (**Figure 4**). LSEC-induced antigen-specific tolerance could also be confirmed in a CD8 T cell-driven acute cholangitis model, both in healthy and fibrotic livers (**Figure 5**). Thus, our findings clearly demonstrated that fibrosis did not impair the ability of LSECs to induce antigen-specific tolerance to ingested antigens taken up by means of their scavenger function.

To assess whether these findings obtained in fibrosis models in mice could be transferable to human liver fibrosis, we reanalyzed published human single-cell sequencing data (31) in comparison to published murine single-cell sequencing data (20), indicating that liver cirrhosis only marginally influenced the expression of scavenger receptors, adhesion molecules and tolerance-related molecules by LSECs both in humans and mice (**Figure 6**). Since we could demonstrate that the marginally altered expression of these molecules in mice did not compromise LSEC tolerance, one can assume that the likewise marginal changes in human LSECs also will not compromise LSEC functionality. Our findings are in line with the study by Su et al. showing conserved LSEC zonation and a general conservation of LSEC identity in cirrhosis (20). Somewhat discrepant findings were obtained in a human single-cell sequencing study comparing normal and cirrhotic liver-derived non-parenchymal cells, reporting a significant reduction of endothelial cells with a normal LSEC signature in cirrhotic livers (24). However, that study was based on isolation of liver non-parenchymal cells. Thus, a reduced number of singlet LSECs might just reflect the particular difficulty of dissociating LSECs from fibrotic livers to singlet cells. Either way, even if LSEC numbers were reduced in fibrosis, LSEC tolerance can still be maintained, as demonstrated by effective protection from autoimmune disease using LSEC-targeting nanoparticles *in vivo* (**Figures 3**, **4**).

Our findings are of direct relevance for the application of nanomedicine products that are currently in clinical translation (12, 42). Previously, it was found that larger microparticles targeting Kupffer cells were also effective in inducing antigen-specific tolerance; however, in liver fibrosis, administration of these particles induced a pro-inflammatory response and disease exacerbation, owed to the plasticity of myeloid cells (43). Given that the prevalence of liver fibrosis in the general population is about 5%, but in some populations also up to 30% (44), clinical application of such Kupffer cell-targeting microparticles might thus cause safety concerns. LSECs reportedly manifest lower plasticity than Kupffer cells, and even ligation of pattern-recognition receptors does not impair their robust tolerance functions (45). Our demonstration that fibrosis also does not impair LSEC tolerance thus indicates that LSEC-targeting nanomedicine products might have a favorable safety profile facilitating their translation into the clinics. Our study also indicates that the scavenger and tolerance function, which is of vital importance to the organism, remains intact in the majority of LSECs in liver fibrosis. It is thus plausible and in line with current understanding that cirrhosis-associated immune dysfunction, which is marked by systemic inflammation and loss of tolerance, is driven by myeloid cells rather than by LSECs (23). How exactly myeloid cells can bypass LSEC tolerance in that condition is not entirely clear, but it appears to be driven by increased microbial translocation from the gut (6) that is associated with this condition (23), and the formation of monocyte-derived syncytia bypassing the sinusoids in advanced cirrhosis (46).

## Data Availability

The original contributions presented in the study are included in the article/**Supplementary Material**. Further inquiries can be directed to the corresponding author.
