# Supplementary material for: Antigen-specific tolerance and control of autoimmunity effected by liver sinusoidal endothelial cells is unimpaired in liver fibrosis
Source: Front Immunol. 2026 May 5;17:1834595. doi: 10.3389/fimmu.2026.1834595 (PMC13183527; doi:10.3389/fimmu.2026.1834595)
Supplement: Supplementary file 1 [file DataSheet1.pdf]

Supplementary Fig. S1

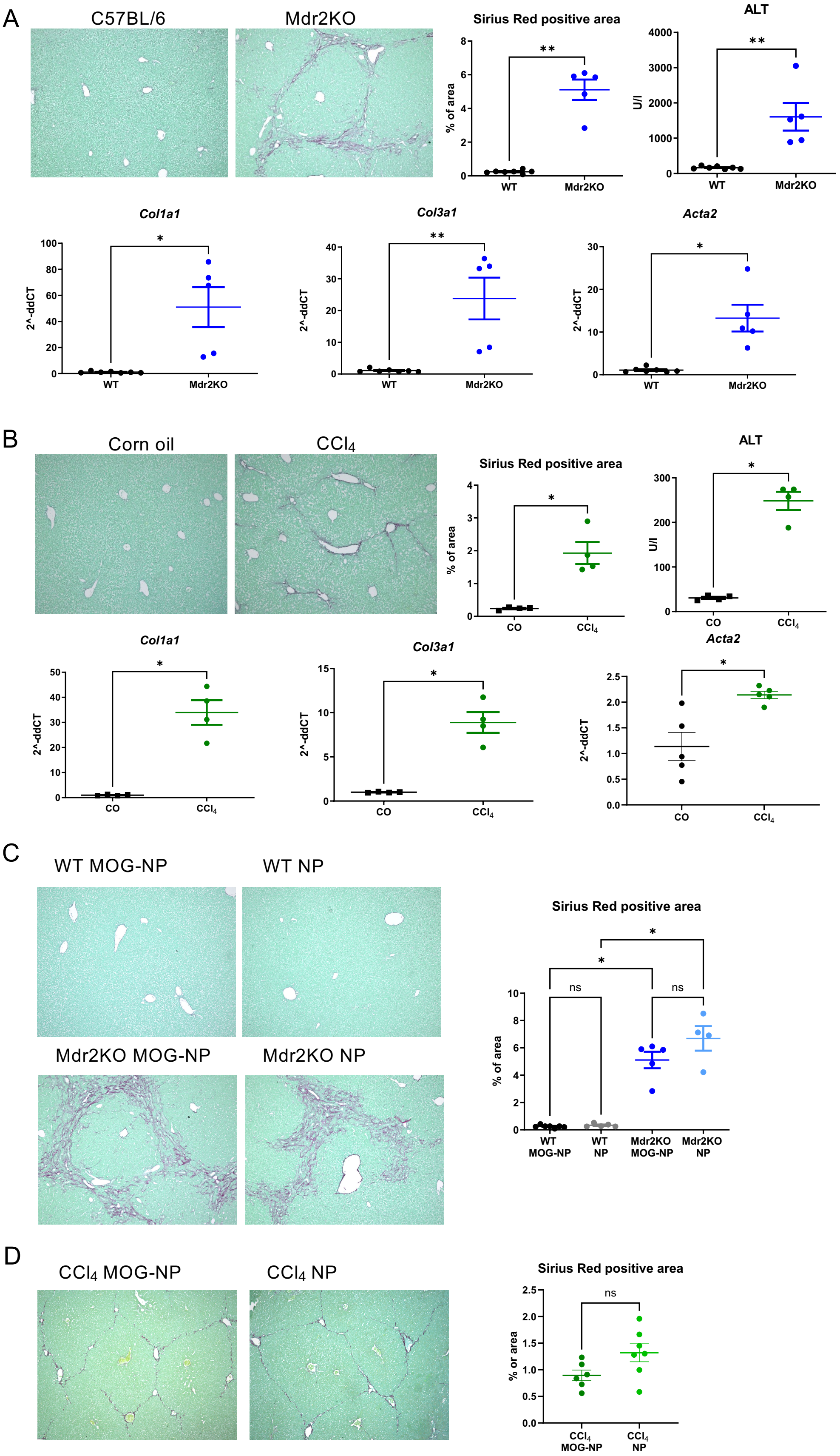

Supplementary Fig. S1: Mouse models of liver fibrosis. (A) Liver fibrosis in Mdr2-knockout mice, as evidenced by Sirius Red staining of representative liver sections, elevated serum ALT levels, and expression of fibrosis-related genes (*Col1a1, Col3a1, Acta2*) in comparison to non-fibrotic C57BL/6 control mice. (B) Carbon tetrachloride (CCl_4)-induced liver fibrosis in C57BL/6 mice, as evidenced by Sirius Red staining of representative liver sections, elevated serum ALT levels, and expression of fibrosis-related genes (*Col1a1, Col3a1, Acta2*) in comparison to corn oil (CO)-treated control mice. (C,D) Confirmation of residual fibrosis 30 days after EAE induction in the Mdr2-knockout model (C) and the CCl_4-induced model (D), both as representative sections and quantitation of Sirius Red. ns = non-significant; * P < 0.05; ** P < 0.01.

Supplementary Fig. S2

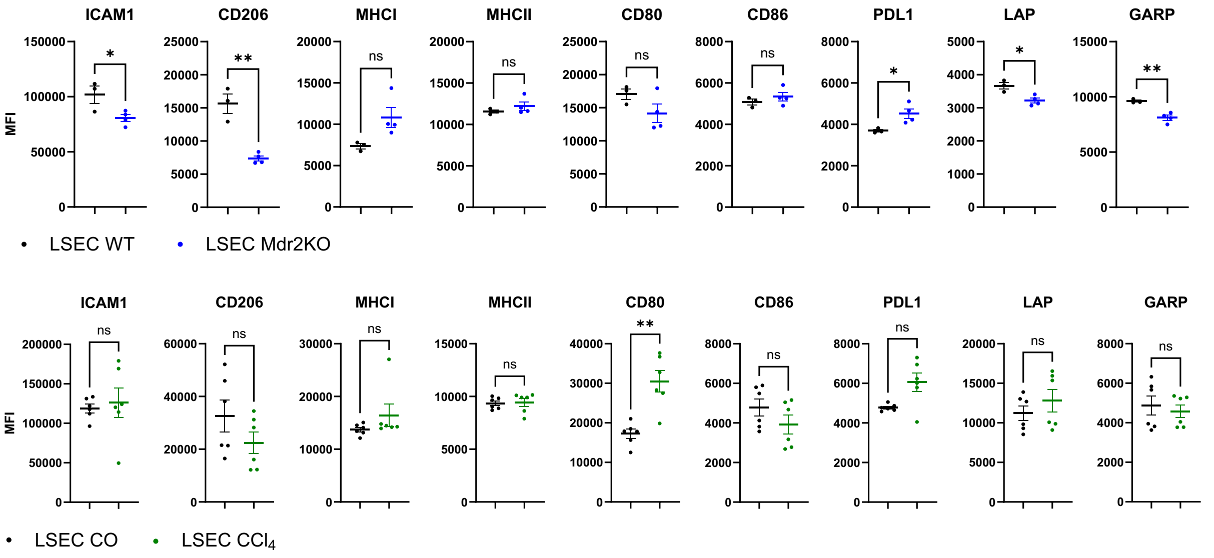

Supplementary Fig. S2: Liver sinusoidal endothelial cell (LSEC) immune molecules detected by flow cytometry. Quantification of protein expression as mean fluorescence intensity (MFI) of selected proteins associated with scavenging, adhesion, or tolerance by LSECs isolated from livers of fibrotic Mdr2-knockout mice (upper row) or from fibrotic livers of carbon tetrachloride-treated mice (lower row) each compared to non-fibrotic control LSECs. ns = non-significant; * $P < 0.05$; ** $P < 0.01$.

Supplementary Fig. S3

A

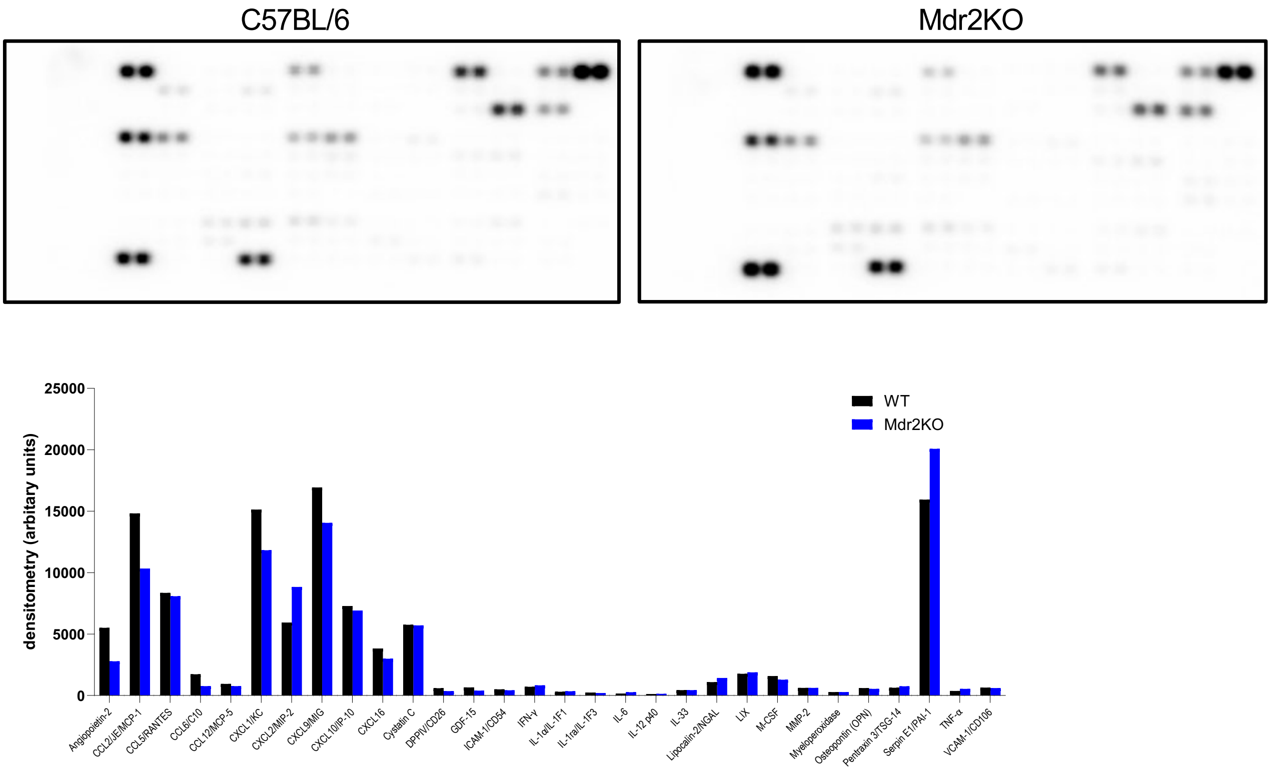

B

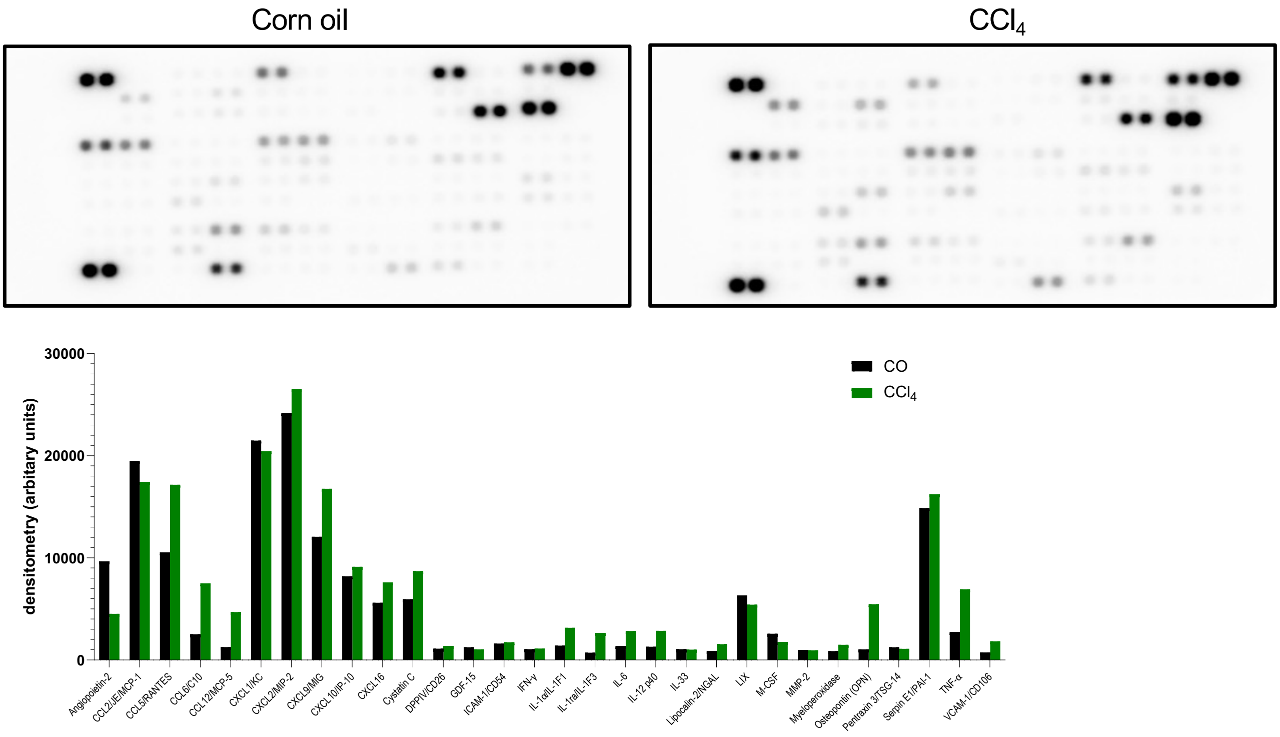

Supplementary Fig. S3: LSEC response to stimulation with inflammatory mediators. LSECs from non-fibrotic or fibrotic livers were isolated by magnetic separation with CD146 beads and taken into culture for 24h, followed by another 24h of stimulation with LPS (10µg/ml) and IFNγ (10ng/ml). The supernatants were analysed with a Proteome Profiler Kit (Mouse XL Cytokine Array). (A) Comparison of LSEC supernatants from non-fibrotic C57BL/6 mice (upper left) and fibrotic *Mdr2*-knockout mice (upper right), and quantification of spot density by densitometry (lower). (B) Comparison of LSEC supernatants from non-fibrotic (corn oil-exposed) and fibrotic (carbon tetrachloride-exposed) C57BL/6 mice (upper row), and quantification of spot densisty by densitometry (lower row).
